# The Administration of an Expectation Survey at a Pain Medicine Clinic to Improve Patient Satisfaction: A Prospective Study

**DOI:** 10.1007/s11916-025-01406-y

**Published:** 2025-07-30

**Authors:** Emmanuella Borukh, Phuong Nguyen, Geum Yeon Sim, Jasal Patel, Andrew Bloomfield, Sarang S. Koushik, Jagun Raghavan, Omar Viswanath, Kevin Zacharoff, Kateryna Slinchenkova, Karina Gritsenko, Naum Shaparin

**Affiliations:** 1 Yeshiva University, New York City, NY USA; 2 Department of Physical Medicine and Rehabilitation, Montefiore Medical Center/Albert Einstein College of Medicine, Bronx, NY USA; 3 Valleywise Health Medical Center, Creighton University School of Medicine, Phoenix, AZ USA; 4 The Ohio State University, Columbus, OH USA; 5 Mountain View Headache and Spine Institute, Creighton University School of Medicine, LSU Shreveport Health Sciences Center School of Medicine, Shreveport, USA; 6 Department of Family, Population, and Preventive Medicine, Renaissance School of Medicine at Stony Brook University, Stony Brook, NY USA; 7 New York Medical Collegem, Valhalla, NY USA; 8 Department of Anesthesiology, Regional Anesthesia & Acute Pain Medicine Fellowship Program, Montefiore Medical Center/Albert Einstein College of Medicine, Bronx, NY USA; 9 Department of Anesthesiology, Multidisciplinary Pain Program, Montefiore Medical Center/Albert Einstein College of Medicine, Bronx, NY USA

**Keywords:** Pain management, Expectation, Satisfaction, Quality improvement, Expectation survey

## Abstract

**Purpose of Review:**

Patients’ expectations are important aspects to consider for improving patients’ satisfaction and willingness to return for continued care. While expectation surveys are not novel in Pain Medicine, none specifically aim to improve satisfaction. This study evaluates whether administering an expectation survey during an initial pain clinic visit improves satisfaction with treatment plans and outcomes. We hypothesized that completing the survey could increase awareness and help align expectations and satisfaction.

**Recent Findings:**

This study was conducted at an outpatient multidisciplinary pain clinic at an urban academic hospital and 100 first-time, English speaking adult patients were recruited. Fifty patients completed a pre-visit questionnaire on pain and expectations (intervention group), while 50 did not (control group). A follow-up survey was completed six months later by 85% of participants to assess satisfaction level with pain treatment, meeting of goals and expectations, and overall clinic experience. No significant differences were found between intervention and control groups for pain treatment satisfaction (3.46 ± 1.31 vs. 3.50 ± 1.28, *p* = 0.48), goal achievement (3.76 ± 1.14 vs. 3.49 ± 1.20, *p* = 0.30), or overall experience (3.83 ± 1.20 vs. 3.72 ± 1.14, *p* = 0.67). Dissatisfaction stemmed from inadequate pain relief, lack of follow-up, and unmet expectations.

**Summary:**

The lack of statistical significance suggests that merely assessing expectations without patient education or provider engagement may be insufficient. Future studies could explore how patient education, communication, and treatment understanding can impact satisfaction to potentially improve pain management experiences.

## Introduction

Patients with chronic pain are inherently more difficult to satisfy [1]. Chronic pain, by definition, develops through a multifactorial process, which makes its complete alleviation nearly unattainable, in contrast to other pathologies of discernible or removable causes [1]. Pain management usually involves a multidisciplinary approach but may still be unsuccessful in abating pain. Chronic pain patients, who frequently expect complete elimination of their symptoms when they visit the Pain Clinic [1], may view this as a therapeutic failure, leading to high dissatisfaction rates and loss of follow-up [2].

Patient satisfaction is defined as the extent to which patients'actual experiences align with their expectations [3]. Notably, high levels of satisfaction contribute to improved treatment adherence and serve as a crucial driver for quality enhancement within the healthcare domain [4]. Various expectation surveys have been developed to gauge and optimize patient satisfaction, with positive outcomes observed in domains like orthopedic joint replacements and dental prosthetic treatments [5, 6].

Although surveys assessing patients’ expectations are not novel in Pain Medicine [7], a notable gap exists in the availability of a survey specifically designed to assess patients'expectations before initial evaluation by Pain Medicine specialists. Expectation surveys during the initial visit before a pain management specialist are an important step in assessing patient goals, establishing realistic expectations, and individualizing treatment plans. When patients’ expectations and preferences are understood and addressed, it can lead to higher satisfaction with the treatment and better engagement in their own care [8]. The present study aims to investigate the potential benefits of administering an expectation survey during the initial pain clinic visit. The study seeks to determine whether the utilization of such a survey would result in increased patient satisfaction, specifically concerning the treatment plan and overall outcome within the context of interventional pain management.

## Methods

This IRB-approved, prospective, controlled study was conducted in an outpatient pain medicine clinic at Montefiore Medical Center Multidisciplinary Pain Center. This study’s protocol and consent documents were reviewed and approved by the Albert Einstein College of Medicine Institutional Review Board (Einstein IRB) on 10/06/2021 under IRB#2021–13115. The study was conducted in accordance with the Declaration of Helsinki.

**Fig. 1:**
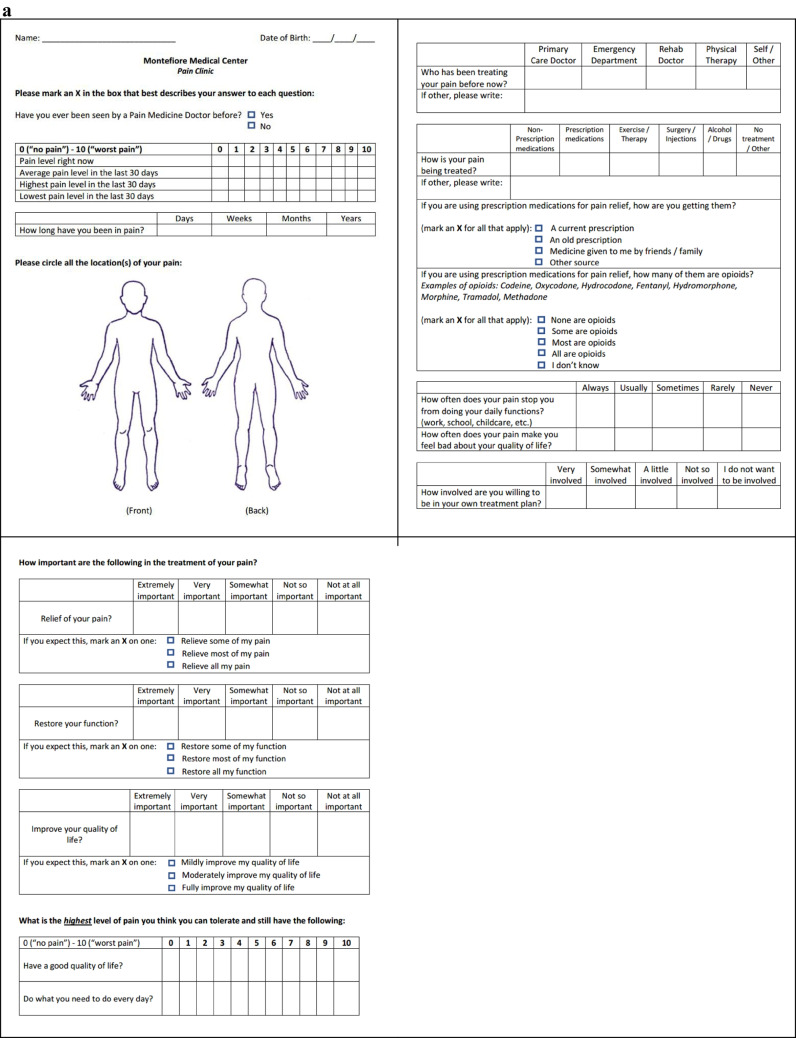

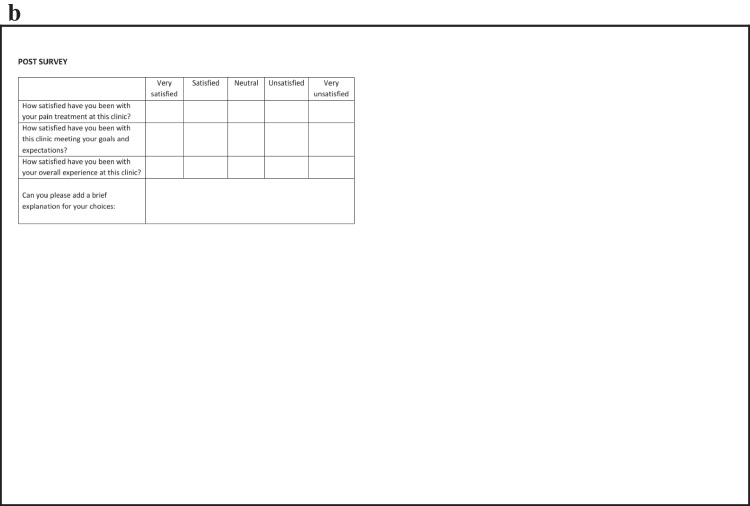
**a**: Sample Initial Questionnaire **b**: Sample Follow-Up Questionnaire

A total of 100 patients were enrolled in the study and split evenly between the control and interventional group. The interventional group received a survey of expectations (Fig. 1a) in the waiting room prior to seeing their providers on the initial visit. Both groups received a follow-up survey (Fig. 1b) assessing their satisfaction via telephone six months after their intake appointment. While the initial protocol called for an in-person administration of the follow-up survey, it was amended to remote follow-up as some patients did not have follow-up appointments. Participants who could not be reached via telephone after 5 attempts were listed as lost to follow-up.

The initial questionnaire served as a survey of expectations and inquired about the duration, severity, prior and current pain treatment (including opioid utilization), and impact of pain on daily function and quality of life. Additionally, patients were asked to rate the importance of pain relief, functional restoration, and improvement of quality of life. The follow-up questionnaire asked patients to rate their satisfaction as it relates to treatment, the clinic meeting their goals and expectations, and their overall experience with the clinic. Satisfaction scores were measured using a Likert scale with scores of 1–5 corresponding to “very unsatisfied,” “satisfied,” “neutral,” “satisfied,” and “very satisfied”, respectively. Participants also had the option to provide comments explaining their answer choices. These comments were later categorized for common themes as they relate to treatment, interactions with providers, and follow-up care.

Since there were no established questionnaires for interventional pain, the questionnaire utilized in this study was designed by the study staff. Overall, the questionnaires aim to gather objective and subjective data regarding the patient's pain condition, treatment goals, and satisfaction levels.

The criteria for inclusion were for patients to be new to the pain clinic, native English speakers, at least 18 years of age, and able to give informed consent. One hundred patients were enrolled prior to their intake appointments. Patients were informed that participation was voluntary, without compensation, and did not affect the standard of care. Written, informed consent was obtained from eligible patients who decided to participate. There were no additional exclusion criteria.

Statistical analysis was performed using Excel and 2-tailed t-tests were used to determine statistical significance. Other statistical values, such as the averages and standard deviations, were determined under basic distributions.

## Results

**Fig. 2:**
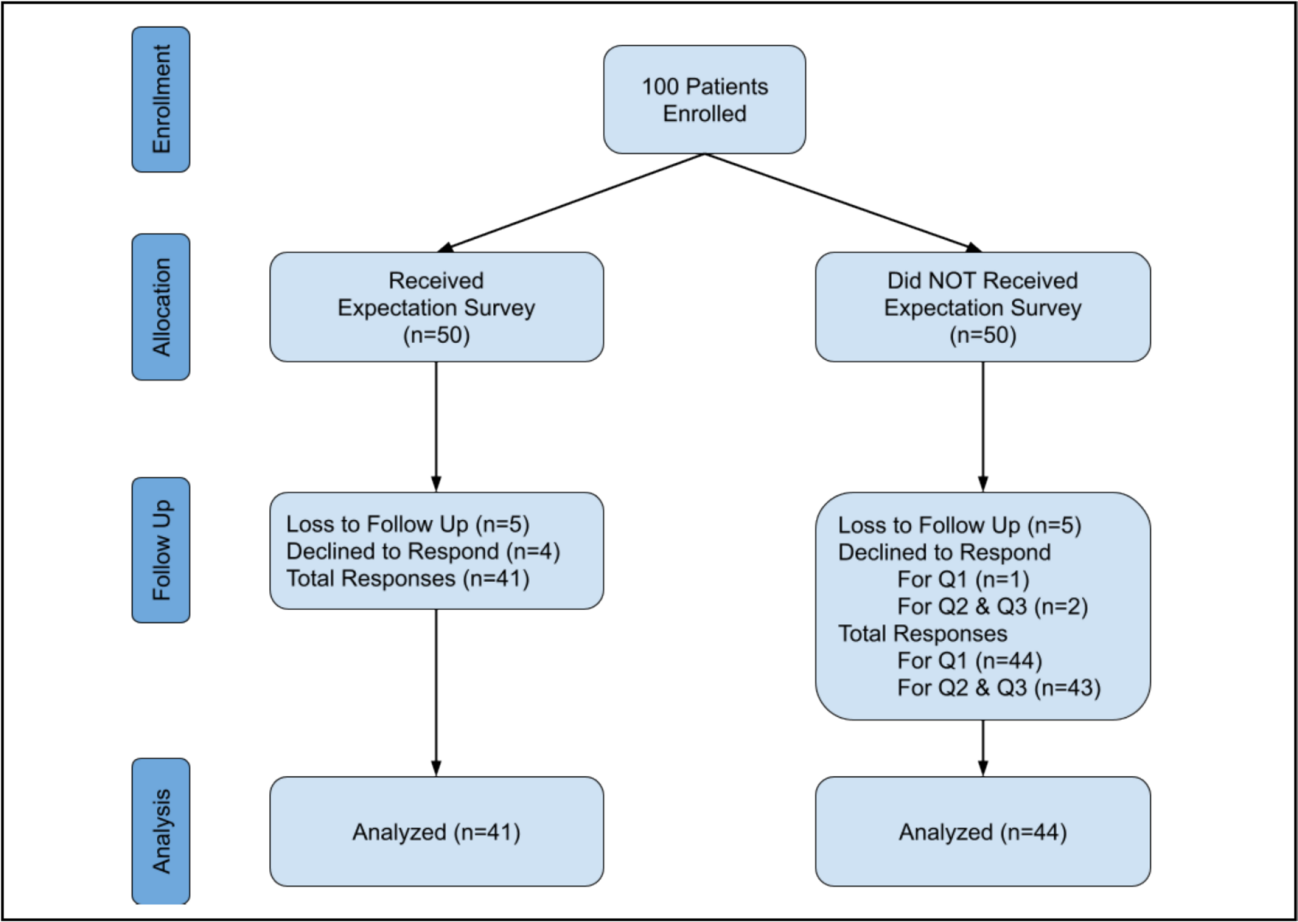
CONSORT Flow diagram of patient enrollment, allocation, and analysis

**Fig. 3:**
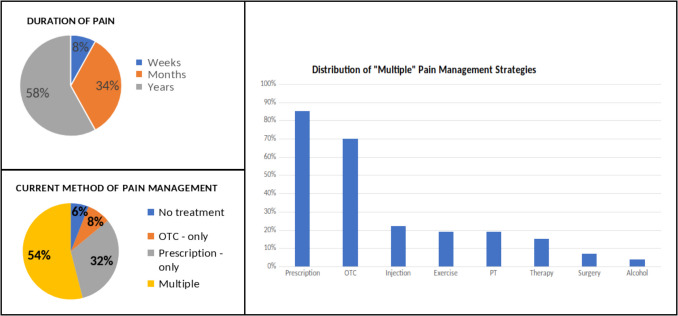
Background of pain length and management at the time of expectation survey administration prior to first appointment

**Fig. 4:**
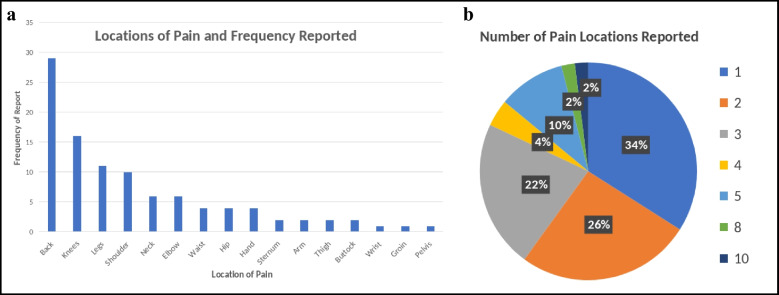
**a** (bar graph) and **b** (pie chart): Reported pain locations in patients receiving initial expectation survey. Multiple patients reported > 1 location of pain, this is reflected in the pie chart on the right

The follow-up survey had an 85% response rate. Of the 15% without responses, 5 participants declined (4 members in the intervention group and 1 member in the control group) and 10 participants were labeled as lost to follow-up (5 members in each group). One participant also chose to only answer the first question from the follow-up survey and declined the remaining questions. This response was included in the analysis (Figs. 2, 3, 4 and Tables 1, 2, and 3).

**Table 1:** Average pain scores at the time of expectations survey administration prior to the first appointment

	**Average (STD)**
Current Pain	7.08 (2.75)
Average Pain	8.64 (1.64)
Highest Pain	9.36 (1.37)
Lowest Pain	6.12 (2.65)

**Table 2:**
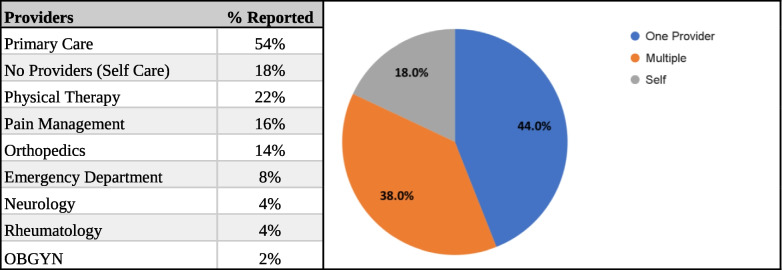
Type of providers patients have seen for pain management. Data based on initial expectation survey

**Table 3:** Satisfaction rating and average score based on follow-up survey responses

	Pain Treatment		Goals & Expectations		Clinic Experience	
	Intervention	Control	Intervention	Control	Intervention	Control
Declined	4	1	4	2	4	2
No Response	5	5	5	5	5	5
Very Unsatisfied	2	4	3	2	3	2
Unsatisfied	12	7	1	8	2	5
Neutral	3	7	11	11	9	8
Satisfied	13	15	14	11	12	16
Very Satisfied	11	11	12	11	15	12
**Total Responses**	**41**	**44**	**41**	**43**	**41**	**43**
**Average Score**	**3.46**	**3.50**	**3.76**	**3.49**	**3.83**	**3.72**
**Standard Deviation**	**1.31**	**1.28**	**1.14**	**1.20**	**1.20**	**1.14**

No statistical significance was observed when evaluating satisfaction scores between the two groups (*p* = 0.30–0.67, Table 4). Mean satisfaction scores were similar between the intervention and control groups, respectively, for pain treatment (3.46 ± 1.31 vs. 3.50 ± 1.28, *p* = 0.48), meeting goals and expectations (3.76 ± 1.14 vs. 3.49 ± 1.20, *p* = 0.30), and overall clinic experience (3.83 ± 1.20 vs. 3.72 ± 1.14, *p* = 0.67). These results indicate that administering the expectation survey did not significantly impact patient satisfaction (*p* > 0.05 for all comparisons, Tables 3, and 4) Figs. 2, 3, and 4.

**Table 4:** 2 tail T-Test analysis of average satisfaction score between intervention vs control group

T- Test Analysis for Follow-Up Questions	*p*-value
How Satisfied Have You been with Your Pain Treatment	0.48
How Satisfied Have You Been with This Clinic Meeting Your Goals and Expectations	0.30
How Satisfied Have You Been with Your Overall Experience at this Clinic	0.67

**Fig. 5:**
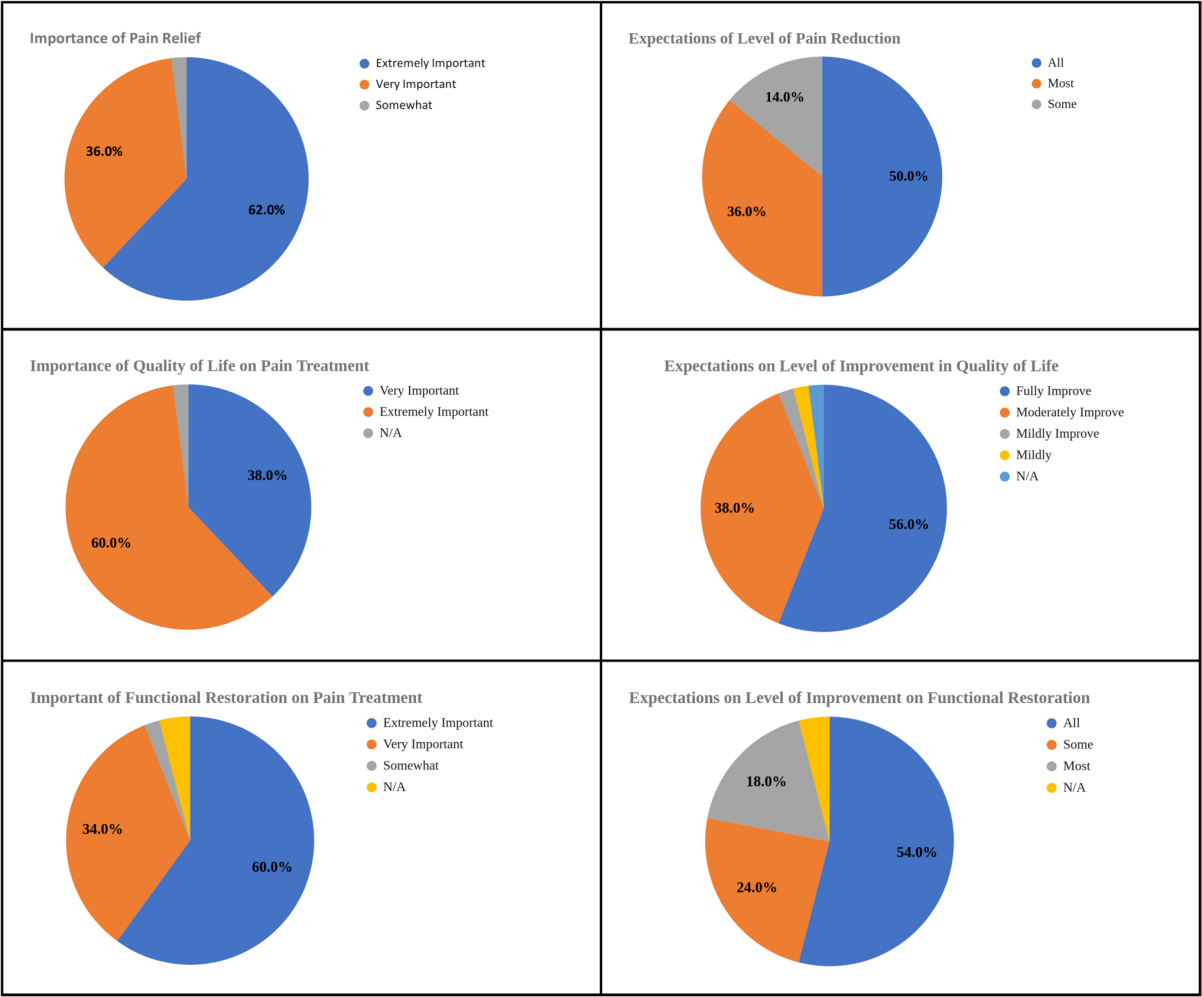
Ratings on level of importance relating to pain treatment, quality of life, and functional restoration. Based on the initial questionnaire

**Fig. 6:**
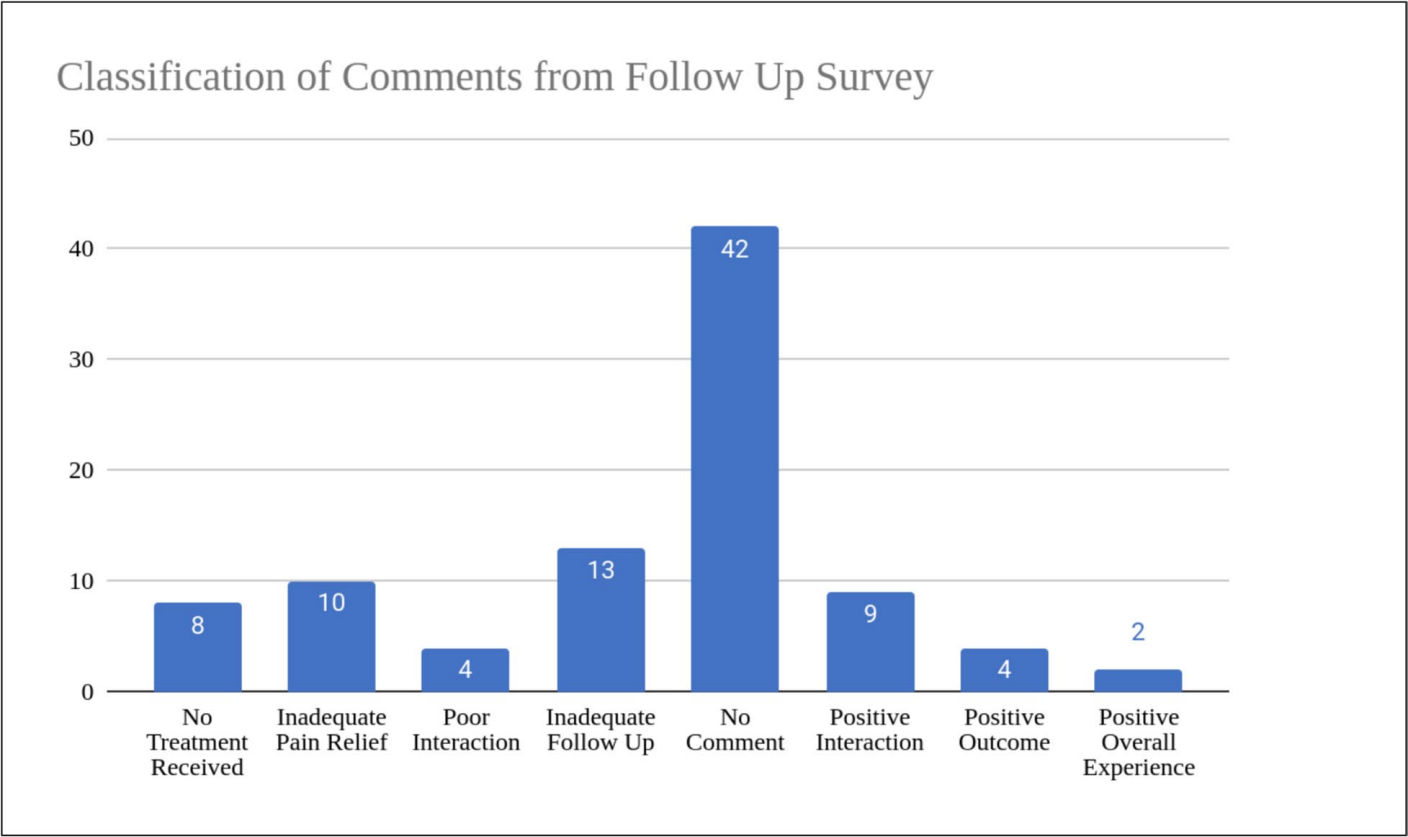
Distribution of comment classification from follow-up survey

**Fig. 7:**
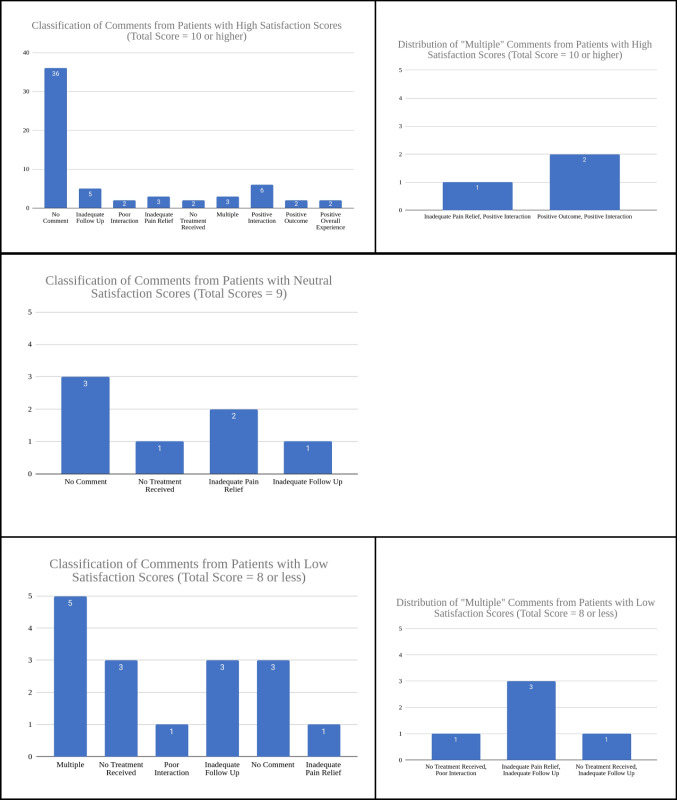
Classification of comments from follow-up survey and comparing the distribution of comments based on participants who gave total satisfaction scores that were low (< 9), neutral (= 9), or high (> 9)

In addition to satisfaction scores, qualitative analysis of patient comments from the follow-up survey (Figs. 5 and 6) provided further insight. Common themes from comments include positive interaction, no treatment received, inadequate pain relief, and inadequate follow-up. Among participants reporting low satisfaction scores (total score < 9), the most frequently cited reasons were inadequate pain relief (11.8%), lack of follow-up (29.4%), and lack of treatment (29.4%). Total scores were calculated by the summation of the Likert scale satisfaction scores of the following three categories: pain treatment, goals and expectations, and clinical experience (Table 3). A score of 9 was deemed neutral, as 3 reflects a neutral standing on the 1 to 5 scale and a 3 in each category totaled to a total score of 9. As such, scores below 9 were considered negative overall, and scores above 9 were considered positive Fig. 7.

## Discussion

Patient feedback surveys are increasingly seen as a key component of monitoring and improving the quality of health care [9]. It is now widely recognized that there is a need for rigorous methods, other than clinical conversations, to elicit patients’ views on such matters as treatment decisions and the quality of care received [10–12]. Our efforts should be directed at making sure that these reports contain reliable and valid indicators of quality and that their focus is on stimulating and guiding quality improvement efforts [10]. Despite no support for the original hypothesis, the initial survey did offer valuable insights into the patient’s experiences with their pain and their expectations when seeking pain management providers. The majority of patients (90%) who came to the clinic reported being in severe pain, with 75% of patients reporting they can still function and have a good quality of life while having moderate to severe pain. Most patients from the intervention group (84%) have never seen pain management previously. All but two patients (96%) wanted to be highly involved in their care and most noted the impact of pain on daily function and quality of life rated as highly important, 94% and 98% respectively. Additionally, about half of the patients expected full restoration and improvement of their function and quality of life. Most patients have had their pain managed by multiple non-pain providers with many being on non-opioid prescriptions.

The study recruited 100 total participants with 15% loss to follow-up. No difference in satisfaction scores was observed between participants who received a pre-visit questionnaire versus those who did not complete the questionnaire. The findings did not support that an expectations survey alone could improve patient satisfaction. Analysis of the average satisfaction of the three follow-up questions pertaining to satisfaction with treatment, expectations met, and overall experience with the clinic revealed minimal differences in rating. The average score for all three questions ranged from 3.49 to 3.83, which corresponded to the nominal rating of “neutral” to “satisfied”. Forty-eight participants provided comments explaining their answer choices and those who rated “neutral” or were not satisfied across all three questions provided more comments compared to those with a higher satisfaction score. Of the lower satisfaction scores, the major complaints stemmed from inept pain relief (11.8%), inadequate follow-up (29.4%), or from patients who did not receive treatment (29.4%).

The current study did not share the participants’ completed expectation survey with their providers because we did not want providers to treat the intervention group differently from the control group. Thus, the survey did not serve as a true intervention to help educate patients and help them set realistic expectations regarding their pain treatments and outcomes. This may have contributed to the lack of noticeable differences in satisfaction scores between the two groups. However, we wanted to explore the direct and psychological impact of the expectation survey on satisfaction without altering the patient-provider interaction between groups.

By providing patients with an expectation survey, patients were forced to reflect on their priorities in pain-related needs. In doing so, another step is added to the patient experience prior to meeting with the provider: introspection. As the patient fills out the survey, they learn about their own expectations and desires regarding their pain clinic visit. We hypothesized that with this additional patient self-awareness, patient experiences would be improved by facilitating patients’ ability to advocate for their priorities and expectations to their healthcare providers. The lack of statistical significance proves that patient self-awareness is not enough to impact satisfaction. The results point to the possibility that it is the relationship between the providers and the patients that would have the most influence over satisfaction rather than independent patient self-reflection. Thus, the lack of valuable difference between the two groups may be due to providers having to be actively involved in personalizing care and educating patients on realistic expectations.

In a recent study by Topan et al., patients who were educated on the rhinoplasty procedure reported higher post-op satisfaction scores than the control group, despite no difference in post-op pain scores between the two groups [13]. This study showed the role that patient education can have in setting expectations and satisfaction of care. While we suggested that patient self-reflection could share a similar result, it is clear providers, not just the patients themselves, need to be made aware of patient expectations and goals. Future studies can explore two deeper routes of intervention. One option is to include an informational section within the questionnaire to educate patients on the types of pain interventions and the level of pain relief patients can expect. Alternatively, the questionnaire could be given to providers who use it to identify patients’ initial expectations, treatment goals, and subsequently engage in further discussion of treatment options and help set realistic expectations. Both of these options could provide valuable insights into the relationship between patient’s expectations and satisfaction scores.

Patient’s satisfaction with their care may depend on the provider’s explanations of treatment goals, patients’ perception of provider engagement, and their understanding of these expectations [14, 15]. As shown in the comment analysis, patient interaction with their provider was a common factor contributing to their satisfaction scores. The follow-up survey in this study did not account for these factors and should be considered in future studies to further assess the impact patient-provider communication has on satisfaction scores, regardless of treatment outcomes.

Additionally, a patient's pain relief can also affect satisfaction [16]. A patient's perception of the degree of pain relief, improvement in daily function, and quality of life at the end of the study could also impact satisfaction scores, which were not assessed in the follow-up survey, and could serve as additional contributing factors. As mentioned prior, inadequate pain relief was a common theme reflected in the comment analysis. Since half of the patients expected full resolution of pain, patient education either pre-visit or during the visit can help set appropriate expectations and affect patient satisfaction. Therefore, measuring these post-treatment ratings in pain relief, function, and quality of life in future studies could be helpful.

Despite the valuable information received, some limitations impacted the conclusions we are able to make. Participants filling out the survey likely assumed that their providers would read their completed expectation surveys and would therefore possibly feel less inclined to advocate for their priorities and desires, assuming the provider would already be aware of them.

We were also able to test the impact of a psychological placebo in relation to the expectation survey. We predicted that since the patients likely assumed that their completed surveys were being read, then they would naturally be more satisfied. However, based on the lack of statistical significance, it is clear no real psychological placebo effect took place.

## Conclusion

Due to the complexity of chronic pain, full resolution of pain is difficult to achieve and creates a challenge in attaining patient satisfaction. Patient dissatisfaction with pain providers can lead to a high rate of turnover in providers and disrupts continuity of care. However, while there are many factors that can affect patients’ satisfaction, there is limited research on patient satisfaction within pain medicine clinics. The current study was a novel approach to exploring whether administering a pre-visit expectation questionnaire would impact post-treatment satisfaction within the scope of interventional pain management. Results showed no significant difference in satisfaction scores among participants who completed a pre-visit questionnaire compared to those who did not. Setting realistic expectations may impact satisfaction. The lack of difference between the two groups showed that patient self-reflection and heightened self-awareness did not produce a measurable positive impact on patient satisfaction, suggesting that meaningful expectation management may require active provider involvement. Additionally, it is important to note that the small sample size likely limited the validity of the study. However, the study also provided insightful trends on the patient's pain experience, initial expectations, and factors affecting satisfaction. While most patients reported severe pain and wanted to be highly involved in their care, they also expected full resolution of pain. Regarding post-treatment satisfaction, the study showed that inadequate pain relief, patient-provider interaction, or lack of treatment play important roles in patient satisfaction. Through these findings, patient education and the patient-provider relationship emerged as a valuable factor that can help set realistic expectations and subsequently impact satisfaction. Further research is needed to evaluate the role of patient education, patient-provider communication, and patient understanding of treatment options as they relate to expectations and overall satisfaction with care.

## Data Availability

Data is provided within the manuscript.
